# A very complicated UTI: malakoplakia following *E. coli* urinary tract infection

**DOI:** 10.1186/s12882-024-03640-9

**Published:** 2024-06-18

**Authors:** Ayotola Fatola, Benjamin C. Johnson, Laura Walsh, Diana Fang, Marissa J. White, Dustin Le, Mohamed G. Atta, C. John Sperati, David N. Hager

**Affiliations:** 1 Department of Medicine, Johns Hopkins University School of Medicine, Baltimore, MD USA; 2 Department of Pathology, Johns Hopkins University School of Medicine, Baltimore, MD USA; 3 Department of Medicine, Division of Nephrology, Johns Hopkins University School of Medicine, Baltimore, MD USA; 4 Department of Medicine, Division of Pulmonary and Critical Care Medicine, Johns Hopkins University School of Medicine, Baltimore, MD USA; 5 Department of Medicine, Johns Hopkins University, Baltimore, MD USA

**Keywords:** Malakoplakia, E. coli UTI, Michaelis-gutmann bodies

## Abstract

Malakoplakia is a rare inflammatory disorder believed to result from a defect in macrophage phagocytic function triggering a granulomatous reaction. It can present with genitourinary, gastrointestinal, or cutaneous manifestations in immunocompromised or, less commonly, immunocompetent hosts. We describe a case of renal malakoplakia in a young, otherwise healthy patient presenting with nephromegaly and sepsis following an *E. coli* urinary tract infection. We discuss diagnosis and management, including antibiotic selection and the decision to pursue nephrectomy. This case highlights the potential for kidney recovery with prolonged antibiotic therapy in conjunction with adjunct immunomodulatory therapies and source control.

## Background

Malakoplakia is an exceedingly rare inflammatory disorder of unknown incidence with approximately 700 cases identified in the literature. It can present with genitourinary, gastrointestinal, or cutaneous manifestations [1]. With renal involvement, patients often have *E. coli* pyelonephritis and acute kidney injury [2]. While *E. coli* is frequently associated with malakoplakia, other gram-negative, gram-positive, and mycobacterial organisms have also been isolated [3]. Malakoplakia occurs most often in patients with weakened immune systems secondary to diabetes, neoplastic disease, autoimmune disease, organ transplant, immunosuppressive therapies, or alcohol use [1, 4]. It is believed to result from a defect in macrophage phagocytic function leading to a diminished bactericidal response [5]. When bacteria are phagocytosed, they are not fully “digested” by macrophages which leads to accumulation of intracellular debris. This in turn triggers a granulomatous reaction [4]. Histologically, this appears as macrophage infiltrates with cytoplasmic inclusions known as Michaelis-Gutmann bodies, the pathognomonic finding in malakoplakia [1, 5]. CD68 and CD163 immunostains confirm the histiocyte lineage, and kidney biopsy typically shows interstitial inflammation with fibrosis and tubular atrophy [6, 7]. Treatment consists of a prolonged course of antibiotics with high intracellular penetration; if medical management is unsuccessful, surgical resection may be required.

### Objective

To highlight malakoplakia as showcased in a young immunocompetent patient, to discuss the pathological findings indicative of malakoplakia, and to review available therapies.

### Case report

A 32-year-old woman with history of recurrent urinary tract infections, Raynaud’s phenomenon, and prior COVID-19 infection presented to the emergency department with lower extremity weakness and 2 days of dyspnea. One week prior, she had experienced symptoms consistent with a UTI but did not pursue testing or treatment. On presentation, she reported nausea, vomiting, fatigue, difficulty walking, and ascending paresthesia in her distal legs. She was afebrile with blood pressure 62/38 mmHg, heart rate 106, and respiratory rate 24. Abdominal examination was notable for hepatomegaly and masses attributed to nephromegaly. A diffuse petechial rash was present, and neurologic exam noted 4/5 strength in the bilateral lower extremities with absent deep tendon reflexes throughout. Although fully alert and oriented on presentation, she became progressively altered. Labs were notable for leukocytosis (WBC 22.22 K/UL), anemia (Hgb 8.0 g/dL), thrombocytopenia (PLT 18 K/UL), elevated inflammatory markers, and kidney failure (creatinine 6.4 mg/dl) with profound hyponatremia and hypokalemia. Blood and urine cultures grew *Escherichia coli.*

**Fig. 1:**
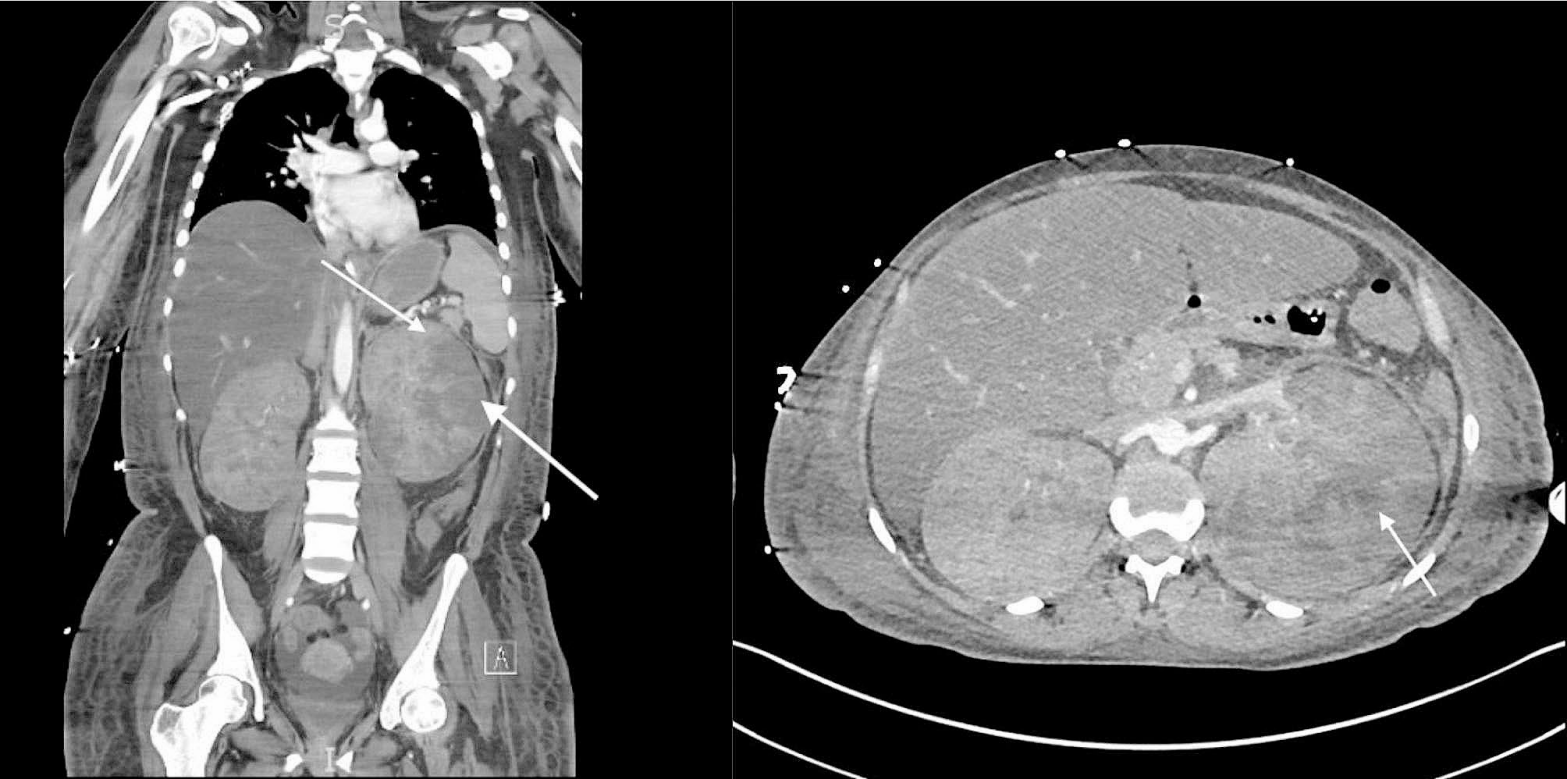
Computed tomography scan of the abdomen/pelvis with intravenous contrast. Massive nephromegaly is present with areas of hypoperfusion and/or infarct (arrows)

Computed tomography scan of the abdomen/pelvis with intravenous contrast revealed massive bilateral kidney enlargement without nephrolithiasis, as well as hepatomegaly (Fig. 1). Initial imaging noted concern for renal vein thrombosis, but no thrombus was seen on subsequent duplex ultrasound. MRI brain and lumbar puncture were performed due to altered mental status and were normal. Despite broad-spectrum antibiotics, her clinical condition rapidly deteriorated, requiring escalating vasopressor support, intubation, and initiation of continuous venovenous hemodialysis (CVVHD).

**Fig. 2:**
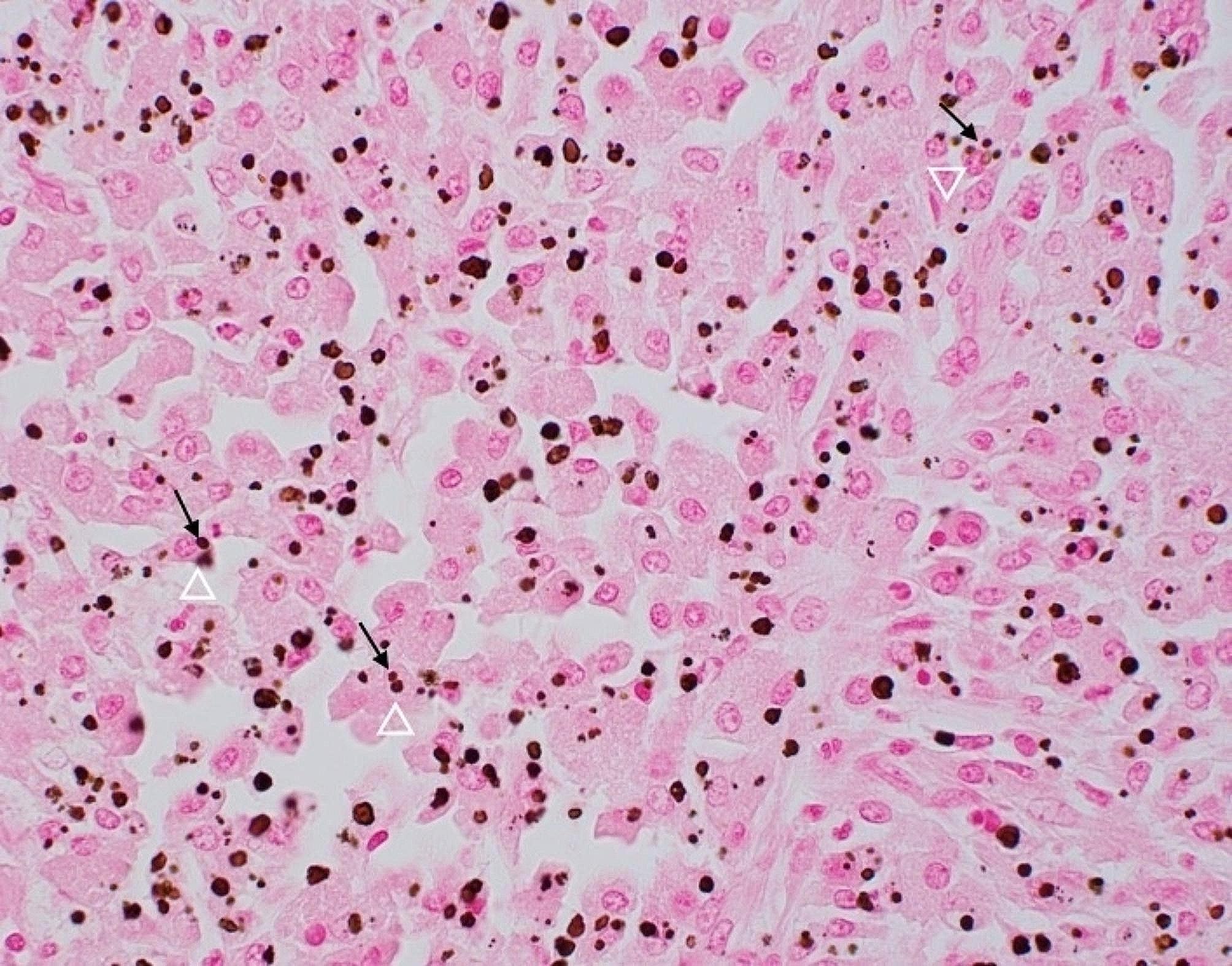
Von Kossa stain of kidney biopsy. Brown cytoplasmic inclusions (arrowhead) within histiocytes (arrow) represent Michaelis-Gutmann bodies, characteristic of malakoplakia

Given the patient’s worsening clinical trajectory and renal function, she underwent percutaneous kidney biopsy. Histopathological analysis demonstrated pyelonephritis with focal micro-abscesses alongside a diffuse histiocytic infiltrate. On light microscopy, only a single ischemic glomerulus was observed, and few tubules were recognizable. Arteries were not identified. Diffusely positive cortical immunohistochemical staining for CD163 confirmed the presence of macrophage/histiocytes, and von Kossa stain was positive in small round formations within the histiocytic infiltrate (Michaelis-Gutman bodies), consistent with a diagnosis of renal malakoplakia (Fig. 2).

Based on prior case reports describing successful treatment of malakoplakia, the infectious disease team recommended changing antibiotics to agents with high intracellular penetration. As the *E. coli* was resistant to fluoroquinolones, trimethoprim, and tetracycline, the patient was started on tigecycline and aztreonam; two weeks later this was changed to minocycline monotherapy. To improve lysosomal function and phagocytosis she was started on ascorbic acid 500 mg daily and bethanechol 10 mg three times daily [6]. Given hepatomegaly, the patient underwent a liver biopsy demonstrating steatohepatitis with moderate macro-vesicular steatosis and abundant Mallory Denk bodies but no evidence of hepatic malakoplakia.

**Fig. 3:**
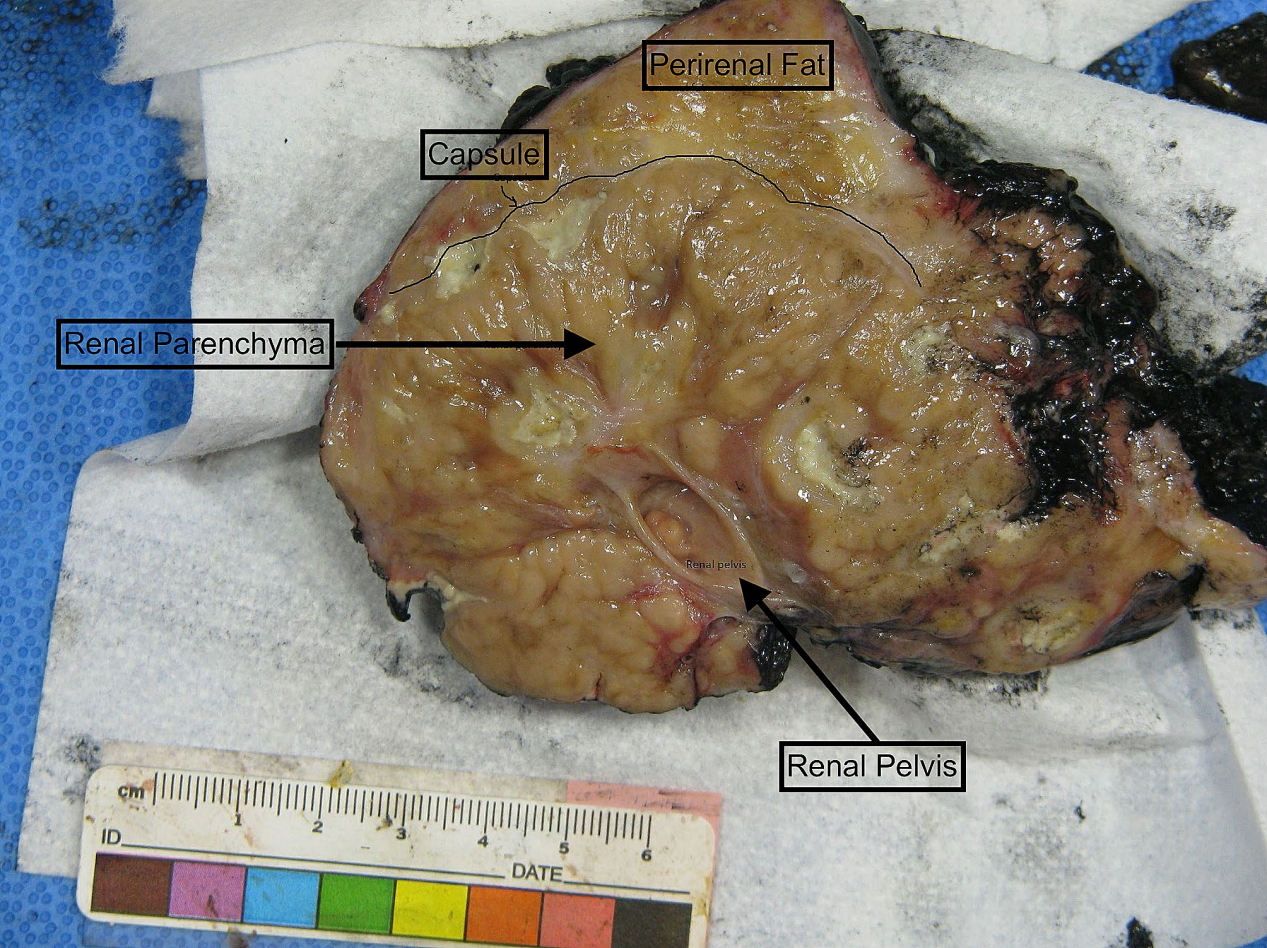
Gross pathology of left kidney and surrounding perinephric soft tissue (overall 13.1 × 10.1 × 8.9 cm), comprised of kidney (8.6 × 6.4 × 8.5 cm) and ureter (not pictured, 5.1 cm in length x 0.4 cm in diameter). The specimen was notable for the extent of inflammation and scarring, as well as tan-yellow nodules with purulent exudate characteristic of malakoplakia

**Fig. 4:**
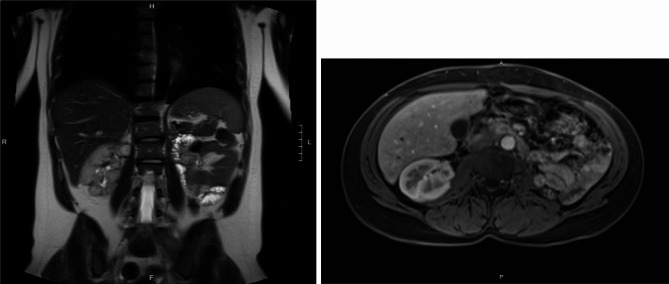
MRI abdomen with and without intravenous contrast performed after 1 year of antibiotic therapy. Imaging is notable for absent left kidney and improvement in right kidney morphology

With treatment the patient’s hemodynamics and kidney function improved. Dialysis was discontinued 31 days after initial presentation. Despite overall clinical improvement, leukocytosis and fevers persisted. Repeat imaging demonstrated enlarging left renal abscesses. A renal mercaptuacetyltriglycine scan on day 45 revealed right and left split kidney function of 75% and 25%, respectively. Given reduced left-sided kidney function with ongoing evidence of infection, a radical left nephrectomy was performed on day 82 (Fig. 3). The patient’s leukocytosis resolved, and she was discharged from the hospital 89 days after initial presentation. Three months after discharge her serum creatinine was 1.2 (eGFR 61 ml/min/1.73m^2^). She completed a 12 month course of oral minocycline, ascorbic acid and bethanechol after repeat MRI demonstrated stable appearance of the right kidney (Fig. 4).

## Discussion

Renal malakoplakia should be considered in patients who present with nephromegaly and urinary tract infections that fail to respond to seemingly appropriate antibiotic therapy. Malakoplakia can occur in patients without known risk factors, as seen in our patient who ultimately underwent whole exome sequencing that showed no reportable variants related to either a primary immunodeficiency or other disease process.

Prompt kidney biopsy provided the diagnosis in this case. Biopsy is required to distinguish malakoplakia from other potential causes of nephromegaly, including diabetes mellitus, cystic disease, and infiltrative diseases such as amyloidosis, lymphoma, and IgG4 related disease. Massive nephromegaly can also be seen in autosomal dominant and recessive polycystic kidney disease, tuberous sclerosis, oro-facial-digital syndrome, and cystic nephroma.

Two unresolved aspects of this case are the patient’s steatohepatitis and neuropathy, which persisted after other symptoms had resolved. Additional workup of the steatohepatitis including alpha-1 antitrypsin, autoimmune studies, viral serologic testing, and phosphatidylethanol testing failed to identify an etiology. Her liver function tests normalized over time. It was postulated that systemic infection may have led to impaired fatty acid oxidation with accelerated triglyceride synthesis leading to acute steatohepatitis. Taken together with the neuropathy, this may suggest our patient had an underlying mitochondrial or fatty acid oxidation disorder not identified through whole exome sequencing.

Given the rarity of malakoplakia, no treatment guidelines exist for this condition [8]. Available literature suggests that a prolonged course of antibiotics with high intracellular penetration such as fluoroquinolones, tetracyclines, or trimethoprim significantly reduces mortality [2, 9]. As in our case, surgical resection should be considered when antimicrobial treatment alone is inadequate [4]. Adjunctive therapies with potential benefit include ascorbic acid to improve lysosomal function and phagocytosis, as well as cholinergic agents like bethanechol to increase the intracellular cGMP to cAMP ratio, since an imbalance between these modulators has been theorized to play a role in defective phagocytosis [6].

The optimal management of severe renal malakoplakia remains undefined. This case may therefore offer guidance in diagnosis, antibiotic selection, adjunctive therapies, and indications for radical nephrectomy.

## Data Availability

Not applicable.
